# Macrophages overexpressing interleukin‐10 target and prevent atherosclerosis: Regression of plaque formation and reduction in necrotic core

**DOI:** 10.1002/btm2.10717

**Published:** 2024-09-16

**Authors:** Mingyi Wang, Shanshan Zhou, Yingyun Hu, Wei Tong, Hao Zhou, Mingrui Ma, Xingxuan Cai, Zhengbin Zhang, Luo Zhang, Yundai Chen

**Affiliations:** ^1^ Medical School of Chinese PLA Beijing China; ^2^ Senior Department of Cardiology The Sixth Medical Center of PLA General Hospital Beijing China; ^3^ Department of Cardiology The First Medical Center of PLA General Hospital Beijing China; ^4^ The Medical School of Nankai University Tianjin China; ^5^ Department of Cardiology No. 966 Hospital of Joint Logisties Force Dandong China; ^6^ The Second Medical School of Southern Medical University Guangzhou China; ^7^ Research Center of Bioengineering The Medical Innovation Research Division of PLA General Hospital Beijing China

**Keywords:** anti‐inflammatory, atherosclerosis, IL‐10, lentivirus, macrophage

## Abstract

Atherosclerosis, a slowly progressing inflammatory disease, is characterized by the presence of monocyte‐derived macrophages. Interventions targeting the inflammatory characteristics of atherosclerosis hold promising potential. Although interleukin (IL)‐10 is widely acknowledged for its anti‐inflammatory effects, systemic administration of IL‐10 has limitations due to its short half‐life and significant systemic side effects. In this study, we aimed to investigate the effectiveness of an approach designed to overexpress IL‐10 in macrophages and subsequently introduce these genetically modified cells into ApoE^−/−^ mice to promote atherosclerosis regression. We engineered RAW264.7 cells to overexpress IL‐10 (referred to as IL‐10M) using lentivirus vectors. The IL‐10M exhibited robust IL‐10 secretion, maintained phagocytic function, improved mitochondrial membrane potentials, reduced superoxide production and showed a tendency toward the M2 phenotype when exposed to inflammatory stimuli. IL‐10M can selectively target plaques in ApoE^−/−^ mice and has the potential to reduce plaque area and necrotic core at both early and late stages of plaque progression. Moreover, there was a significant reduction in MMP9, a biomarker associated with plaque rupture, in IL‐10M‐treated plaques from both the early and late intervention groups. Additionally, the administration of IL‐10M showed no obvious side effects. This study serves as proof that cell therapy based on anti‐inflammatory macrophages might be a promising strategy for the intervention of atherosclerosis.

**Abbreviations**
ConM: control macrophages
IL‐10M: IL‐10‐overexpressing macrophages
IVIS: intravital imaging system
ORO: Oil Red O
KEGG: Kyoto Encyclopedia of Genes and Genomes
GSEA: gene set enrichment analysis
WD: Western diet

**Translational Impact Statement**

In the present study, we overexpressed IL‐10, an anti‐inflammatory protein, in macrophages and subsequently administered these cells to ApoE^−/−^ mice. Leveraging the inherent homing ability of macrophages, IL‐10M was selectively targeted toward atherosclerotic plaques, thereby reducing the plaque area and necrotic core through modulation of the inflammatory microenvironment within them. These data support the possibility of IL‐10M as an alternative immunomodulatory therapeutic strategy to promote atherosclerosis regression and reduce cardiovascular events.

## 1 INTRODUCTION

Persistent cardiovascular diseases (CVD) are significant contributors to global mortality.^1^ Despite the existence of established therapeutic strategies for patients, an inherent risk of recurrent cardiovascular events still exists.^2^ Findings from randomized controlled trials have indicated that, in patients undergoing statin therapy, the residual inflammatory risk is more strongly correlated with future cardiovascular events and all‐cause mortality than the residual cholesterol risk.^3^ Both the Colchicine Cardiovascular Outcomes Trial^4^ and Low‐Dose Colchicine for Secondary Prevention of Cardiovascular Disease 2 Trial^5^ have provided evidence that the anti‐inflammatory medication can effectively reduce cardiovascular events in patients with recent myocardial infarction and chronic coronary heart disease. These findings underscore the therapeutic value of anti‐inflammatory approaches in the treatment of CVD.

Atherosclerosis is a key mechanism for CVD. The distinct tropism of macrophages toward plaques and their involvement in plaque progression presents opportunities for macrophage‐based cell therapy in the context of atherosclerosis. Modifications are essential for optimizing macrophages to achieve desired therapeutic effects. In the present study, lentiviral transfection was employed to generate engineered macrophages. In terms of gene transfer, the lentivirus possesses several unique advantages: stable and persistent expression, ability to infect various cell types, capacity to carry large gene fragments, relatively low immunogenicity, and minimal induction of host immune responses. In a previous study by Liu et al., lentivirus loaded with interleukin (IL)‐10, Cd147, and Tgfrcfc RNAs were used to separately transfect RAW264.7 macrophages. The cells were then injected into mice for the treatment of bleomycin‐induced lung injury.^6^

The anti‐inflammatory cytokine IL‐10 is produced by activated immune cells.^7^ Studies have shown that IL‐10 exerts a clear anti‐plaque effect.^8^, ^9^ IL‐10 knockout mice, in comparison to wild‐type C57BL/6 mice, are more prone to plaque formation. When IL‐10 plasmids were introduced into IL‐10 knockout mice, it resulted in a 60% reduction in the plaque area.^10^ The introduction of the IL‐10 gene into ApoE^−/−^ or Ldlr^−/−^ mice significantly inhibited plaque formation.^11^, ^12^ Clinical trials has been conducted to explore the potential of IL‐10 cytokine therapy for inflammatory diseases, such as rheumatoid arthritis, inflammatory bowel disease and tumors.^13^, ^14^ However, the direct use of IL‐10 monomers as therapeutic drugs has significant limitations. The systemic administration of IL‐10 is associated with a short half‐life, and achieving adequate concentrations at disease sites can be challenging.^15^ Studies have sought to address these limitations by delivering encapsulated IL‐10 within nanoparticles for the treatment of atherosclerosis.^16^, ^17^ Notable efforts have been made to harness the antitumor immunity of IL‐10 through the engineering of an IL‐10/Fc fusion protein. The addition of this fusion protein to either mouse chimeric antigen receptor T cell therapy or immune checkpoint blockade led to remarkable tumor regression.^18^ Based on the unique homing and pivotal role in plaque progression and regression, we aim to harness macrophages in conjunction with IL‐10. On one hand, IL‐10 exerts its anti‐plaque effect, while on the other hand, it can also modulate the macrophage phenotype to create a more favorable microenvironment within plaques. To achieve this goal, we employed a lentivirus harboring Il‐10 mRNA to infect macrophages, resulting in the overexpression and oversecretion of IL‐10.

This study aimed to investigate the feasibility and effectiveness of an approach designed to overexpress IL‐10 in macrophages and subsequently introduce these genetically modified cells into an atherosclerosis mouse model. The findings from this research can aid in assessing how macrophages, one of the most abundant cells in atherosclerotic plaques, can be harnessed as a platform to locally deliver anti‐inflammatory proteins to promote atherosclerosis regression.

## 2 RESULTS

### 2.1 Establishment and characterization of engineered macrophages

In order to build modified macrophages, we utilized RAW264.7 macrophages owing to their broad drug‐delivery applicability. We genetically engineered these cells to create IL‐10M using a lentiviral vector. Control cells (ConM) were generated without the IL‐10 sequence.

**FIGURE 1:**
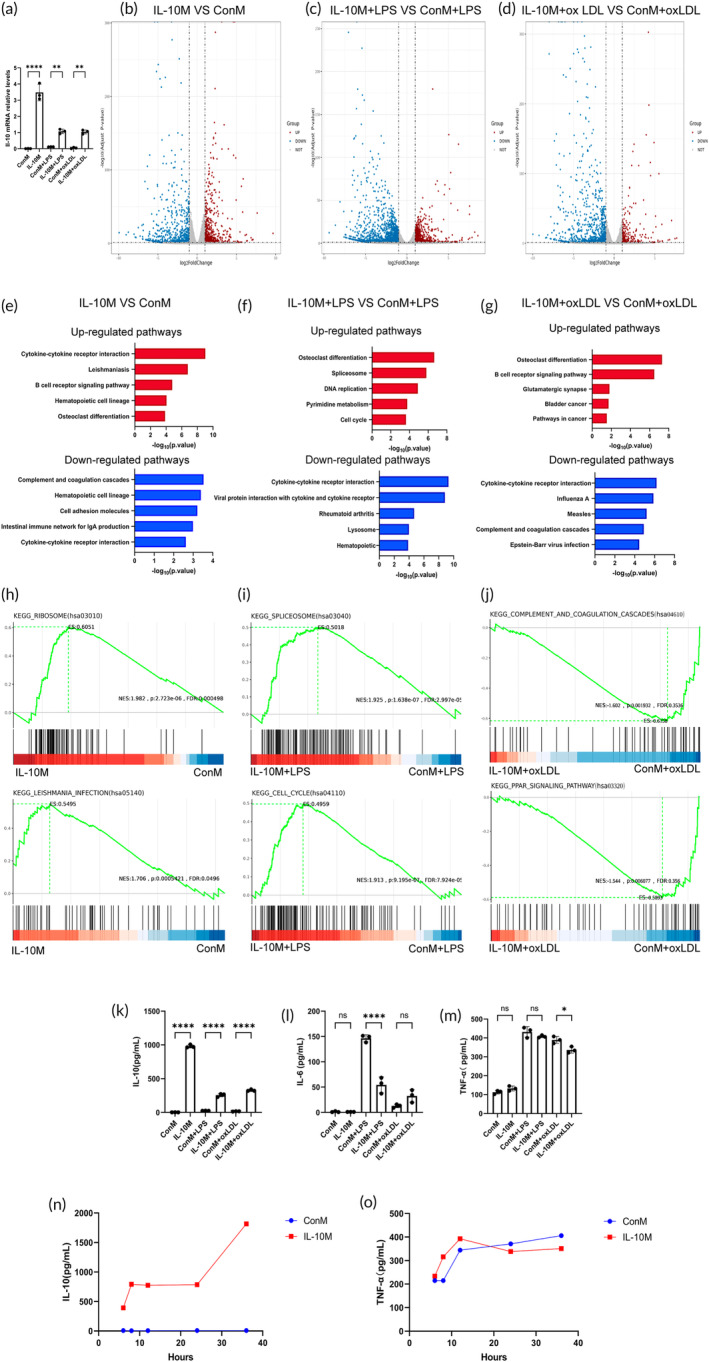
Transcription and protein expression levels of engineered macrophages. (a) Il‐10‐relative mRNA expression levels obtained using RNA‐seq. (b–d) Volcano plots of differentially expressed genes. (e–g) Kyoto Encyclopedia of Gene and Genomes (KEGG) pathway enrichment analysis of differentially expressed genes. (h–j) Gene set enrichment analysis (GSEA) of differentially expressed genes. (k–m) Concentrations of IL‐10, IL‐6, and TNF‐α in the culture medium under different stimulation conditions. (n, o) Concentrations of IL‐10 and TNF‐α measured at various time points in the culture media of IL‐10M and ConM.

Transcriptomic analysis revealed that IL‐10M consistently exhibited significantly higher IL‐10 mRNA levels (Figure 1a), regardless of LPS stimulation, along with the differential expression of 1271 genes in non‐stimulated conditions (Figure 1b). After 12 h of LPS stimulation, 2214 genes were differentially expressed in IL‐10M compared to ConM (Figure 1c). After 24 h of oxLDL stimulation, 1226 genes differed in expression between the IL‐10M and ConM (Figure 1d). The KEGG pathway analysis demonstrated that factors related to the osteoclast differentiation pathway were up‐regulated in IL‐10M, LPS stimulated IL‐10M and oxLDL stimulated IL‐10M (Figure 1e–1g). Genes involved in the osteoclast differentiation pathway including PI3K‐Akt, NF‐κB, MAPK, calcium, and Jak–STAT signaling pathways, which play a crucial role in macrophage polarization. The results of GSEA demonstrated that the genes responsible for the disparities between IL‐10M and ConM are predominantly enriched in the ribosome and leishmania infection related pathways (Figure 1h), thereby further validating successful macrophage infection by IL‐10‐carrying lentivirus. Upon LPS stimulation, differentially expressed genes in IL‐10M are primarily enriched in the spliceosome and cell cycle pathways (Figure 1i), suggesting that IL‐10M may actively modulate protein modifications and regulate cell cycle as a response to inflammatory stimuli. Upon oxLDL stimulation, the discrepancies between IL‐10M and ConM mainly manifest as downregulation of genes associated with complement and coagulation cascades as well as PPAR signaling pathway (Figure 1j), indicating differences in lipid metabolism between IL‐10M and ConM.

We confirmed sustained secretion of high levels of IL‐10 in the culture medium of IL‐10M using ELISA (Figure 1k). After LPS stimulation, IL‐10M exhibited significantly lower IL‐6 expression than ConM (Figure 1l). After oxLDL stimulation, IL‐10M showed significantly lower concentrations of TNF‐α than ConM (Figure 1m). By monitoring the temporal trends in IL‐10 and TNF‐α secretion between IL‐10M and ConM, it was observed that IL‐10M exhibited sustained high expression of IL‐10 after altering the culture medium, while the expression of the pro‐inflammatory cytokine TNF‐α remained essentially consistent with ConM (Figure 1n,o).

**FIGURE 2:**
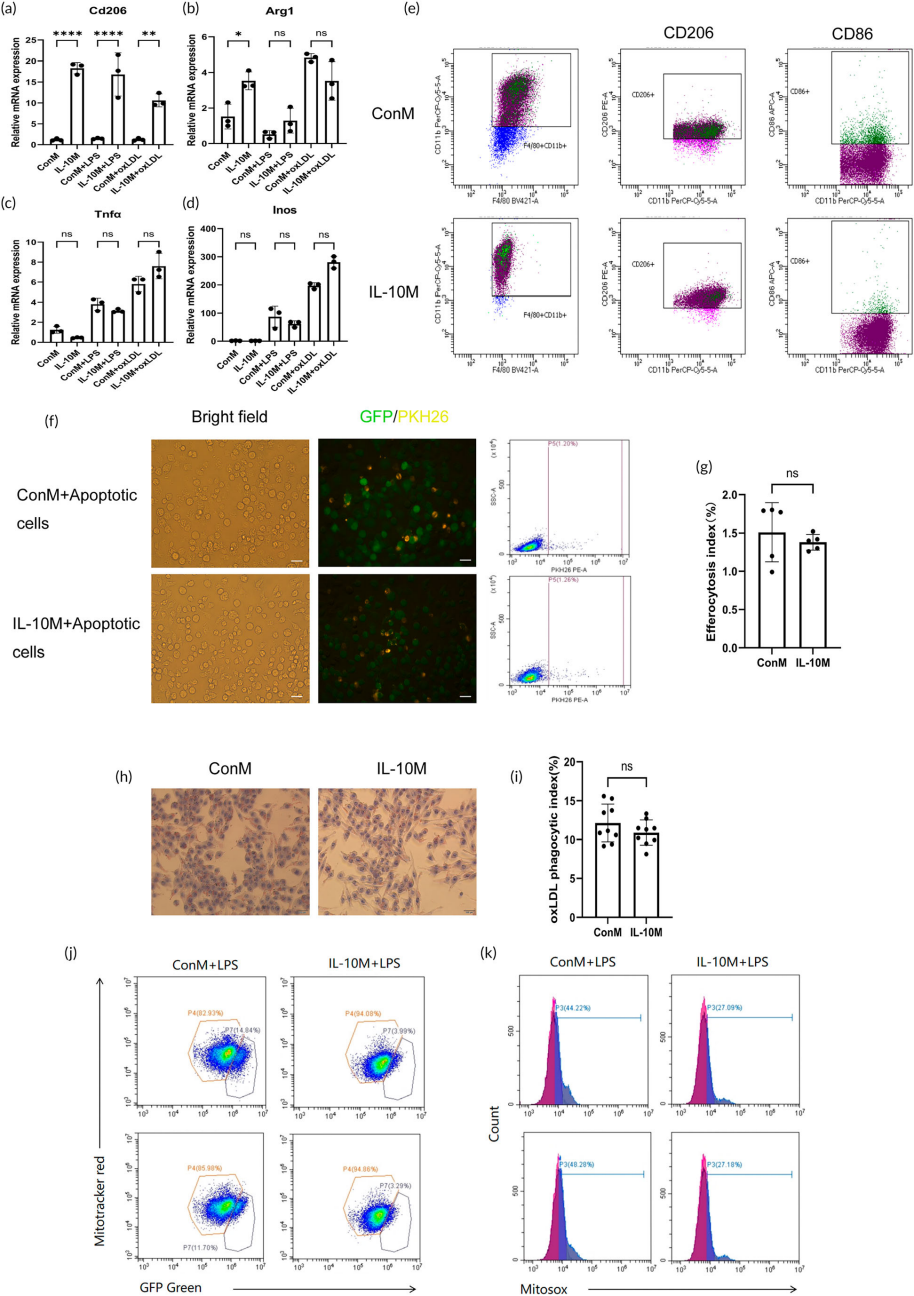
Polarization phenotypes, phagocytic functions and mitochondria activity of engineered macrophages. (a–d) RT‐qPCR was conducted to assess the polarization phenotypes. (e) Flow cytometry was performed to determine the polarization phenotypes. (f) Efferocytosis assay: Fluorescence microscopy revealed engulfment of apoptotic cells by IL‐10M and ConM, with measurement of fluorescence signals using flow cytometry. Both IL‐10M and ConM expressed green fluorescent protein (GFP), while apoptotic cells were stained with the PKH26 dye. Scale bar: 100 μm. (g) Comparison between IL‐10M and ConM in terms of the efferocytosis index (%). (h) Phagocytosis of oxLDL by IL‐10M and ConM was evaluated, where red particles represented oxLDL. Scale bar: 100 μm. (i) Comparison of the phagocytic index (%) for oxLDL between IL‐10M and ConM. (j) Flow cytometry analysis of the mitochondrial membrane potentials in IL‐10M and ConM stimulated with LPS for 12 h. (k) Flow cytometry analysis of the superoxide content in mitochondria stimulated with LPS for 12 h in IL‐10M and ConM.

IL‐10M maintained an anti‐inflammatory M2 phenotype, with a significantly higher mRNA expression of the M2 marker Cd206 than ConM, even after stimulation with LPS or oxLDL (Figure 2a). The mRNA expression of the M2 marker Arg1 was significantly higher in IL‐10M (Figure 2b), whereas there were no significant differences observed in the mRNA levels of the M1 markers Tnf‐α and Inos (Figure 2c,d). Validation through flow cytometry further emphasized the increased expression of CD206 and decreased expression of M1 marker CD86 in IL‐10M (Figure 2e). Efferocytosis and lipid phagocytosis assays indicated that IL‐10M retained the capacity to phagocytose apoptotic cells and lipids (Figure 2f–2i), which is critical for plaque stability. Utilizing MitoTracker Red CMXRos and MitoSOX staining, it was demonstrated that IL‐10M exhibited improved mitochondrial membrane potentials and reduced superoxide production following LPS stimulation (Figure 2j,k).

### 2.2 Targeted delivery of engineered macrophages in atherosclerotic mice

**FIGURE 3:**
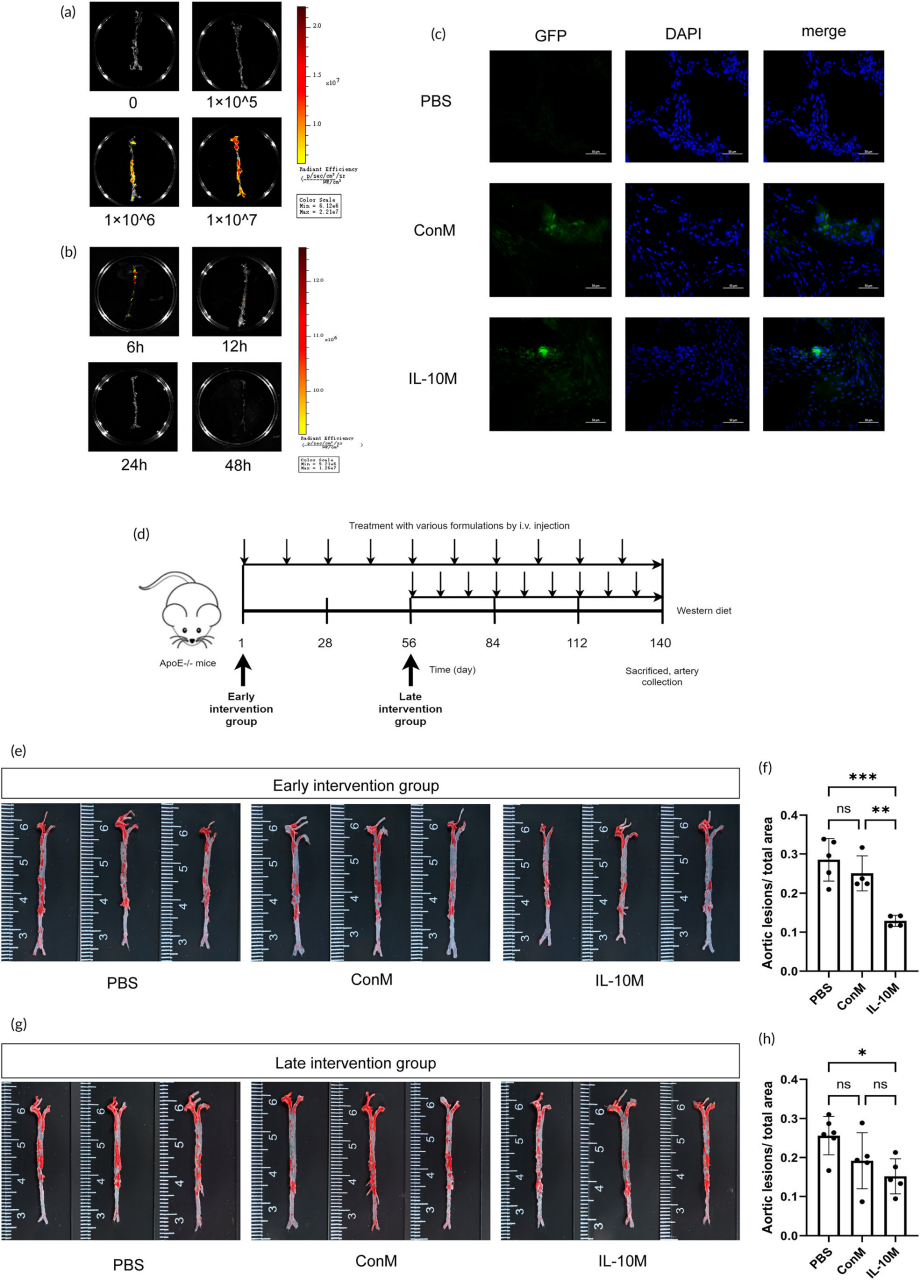
Targeting and therapeutic effect of engineered macrophages on atherosclerotic plaques. (a) Aortic IVIS imaging was conducted after the intravenous injection of varying quantities of IL‐10M in atherosclerotic mice. Isolated aortas were placed in 6 cm dishes. (b) Intravenous injection of 1 × 10^7^ IL‐10M followed by IVIS imaging at different time points. (c) Following the intravenous administration of IL‐10M and ConM in atherosclerotic mice, aortic plaques were isolated, sectioned, stained with DAPI, and visualized using fluorescence microscopy. Scale bar: 50 μm. (d) Flowchart illustrating the intervention protocol. (e) Oil Red O staining results for the early intervention group treated with different formulations in the aorta. (f) Statistical analysis depicting aortic lesions/the total aortic area is shown in panel “e.” (g) Oil Red O staining results for the late intervention group treated with different formulations in the aorta. (h) Statistical analysis depicting aortic lesions/the total aortic area is shown in panel “g.”

We illustrated the capacity of IL‐10M to specifically target atherosclerotic plaques within the aorta by administering IL‐10M via tail vein injection. To confirm the precision of this targeting, IL‐10M was administered via tail vein injection to ApoE^−/−^ mice fed a WD for 3 months. Subsequently, the IVIS was used to assess the fluorescence signals emanating from IL‐10M across the entire aorta. Four separate groups were established, each comprising ApoE^−/−^ mice: one serving as a control and the others receiving injections of 1 × 10^5^ cells, 1 × 10^6^ cells, and 1 × 10^7^ cells, respectively. IVIS imaging was conducted 6 h post‐injection. The results unequivocally demonstrated a positive correlation between cell concentration and fluorescence signals within the aorta (Figure 3a). To further examine signal attenuation, we introduced various time points after the injection of 1 × 10^7^ cells. Our findings revealed a marked reduction in the fluorescence signal 6 h post‐injection (Figure 3b).

Following the injections, the aortas were excised for pathological sectioning and subsequently visualized using fluorescence microscopy. Green fluorescence emanating from IL‐10M was conspicuously observed within the plaque area of mouse arteries, providing further evidence of the exceptional specificity of IL‐10M in targeting plaques (Figure 3c). In addition, ex‐vivo imaging results of major organs, including the heart, liver, spleen, lung, and kidney in mice 6 and 72 h after cell injection are presented in Figure S1. The liver exhibited the strongest fluorescence signal, followed by the spleen. Fluorescence imaging of histopathological sections revealed accumulation of IL‐10M and ConM in both the liver and spleen, with a significant decrease in the positive signal observed at 72 h compared to that at 6 h post‐injection (Figure S2). Detection of IL‐10 concentrations in the aorta and major organs 6 and 72 h after cell injection demonstrated that IL‐10 was primarily secreted in the aorta, liver, and spleen (**Figure** S3a). Moreover, IL‐10 concentrations in serum 6 and 72 h after cell injection were higher in the IL‐10M group (**Figure** S3b). No evident signs of injury were detected in H&E‐stained organs in either treatment group at both time points (**Figure** S4). Based on these findings and previous studies,^6^, ^19^, ^20^ a dosage of 1 × 10^7^ cells administered every 1–2 weeks was selected as the optimal treatment regimen.

### 2.3 IL‐10M reduce plaque area

Subsequently, we validated the effectiveness of IL‐10M in mitigating atherosclerotic burden. ApoE^−/−^ mice were fed a WD to stimulate the development of atherosclerotic plaques. We established early and late intervention groups in the context of IL‐10M intervention for atherosclerosis.

The early intervention group received IL‐10M injections commencing at the onset of WD feeding and continued until week 20, whereas the late intervention group received injections from weeks 8 to 20 following the initiation of WD feeding. Figure S5 illustrates the plaque burden in the aorta of mice in the early and late intervention group prior to injection. At 8 weeks of age, no discernible plaques were observed in the aorta of mice in the early intervention group; however, by 16 weeks of age, significant plaque formation was evident in the aorta of mice in the late intervention group. The early and late intervention groups were further subdivided into three treatment subgroups, each receiving a different injection formulation: IL‐10M, ConM, and PBS.

After harvesting at 20 weeks of WD feeding, the extent of plaque formation was meticulously assessed by ORO staining of the entire aorta (Figure 3d). Our findings unequivocally demonstrated that intervention with IL‐10M elicited a substantial reduction in plaque area after 20 weeks of WD feeding. Specifically, IL‐10M‐treated mice exhibited a significantly diminished plaque area in the aorta compared with mice treated with ConM or PBS in both early and late intervention groups (Figure 3e–h).

### 2.4 IL‐10M enhance plaque stability

**FIGURE 4:**
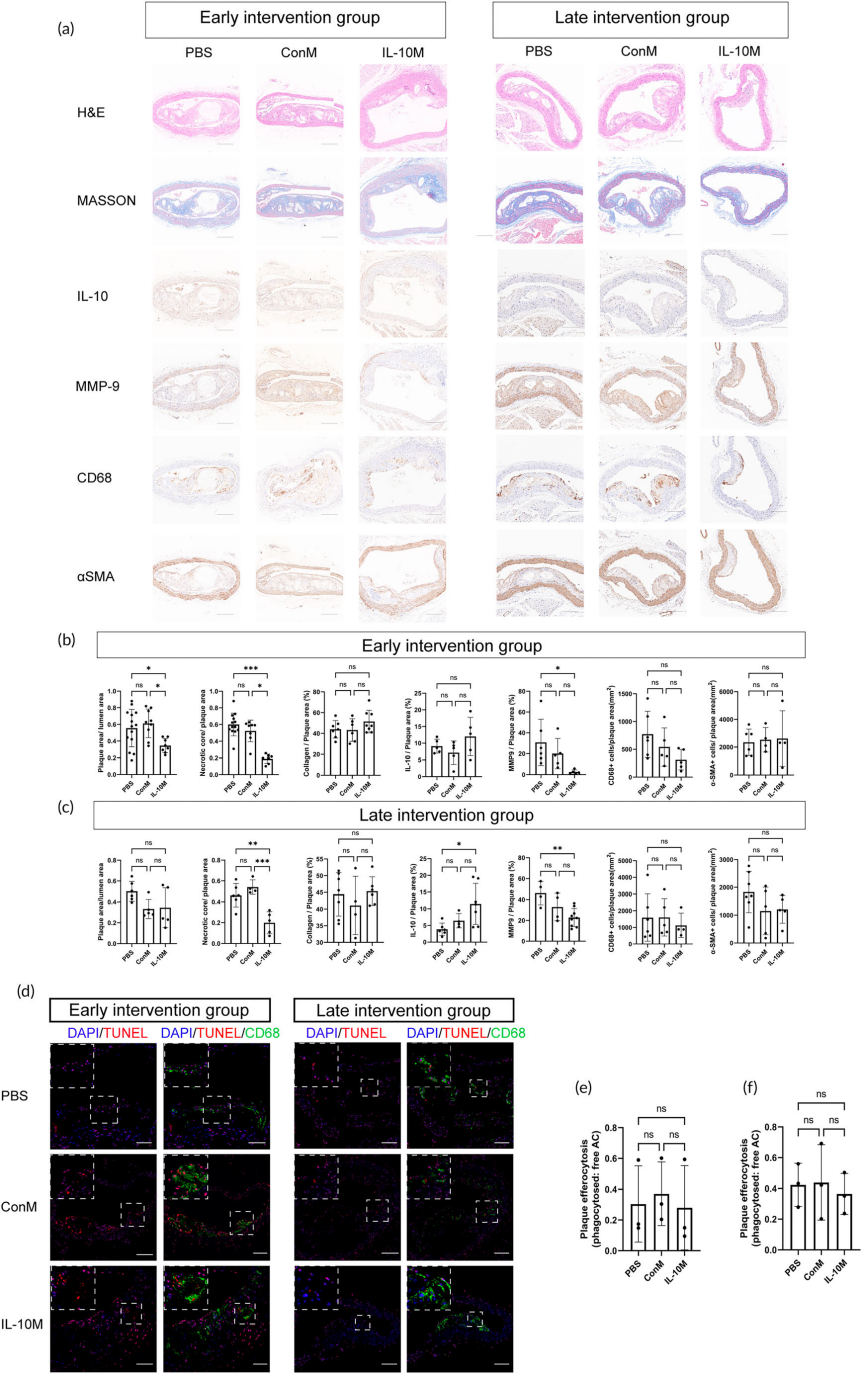
Action mechanism of IL‐10M in the treatment of atherosclerosis. (a) H&E, Masson and Immunohistochemistry of various markers in the plaques after treatment in the early intervention and late intervention groups. Scale bar: 50 μm. (b, c) Statistical analysis regarding each marker shown in panel “a.” (d) Representative images of the in situ efferocytosis assay: Phagocytosis of apoptotic cells (TUNEL‐positive) by CD68‐positive macrophages within the plaques after treatment with different formulations. (e, f) Quantification of the ratio of phagocytosed to free apoptotic cells.

To evaluate the pathological characteristics of aortic plaques following treatment with various formulations, we selected aortic arch plaques for histopathological sectional staining. H&E staining revealed that mice treated with IL‐10M in the early intervention group showed a significant reduction in the ratio of plaque area to lumen area in mice (Figure 4a–c). However, the late intervention group did not exhibit any statistically significant differences (Figure 4a–c). Notably, mice treated with IL‐10M exhibited significantly smaller necrotic cores within the aortic plaques than those treated with ConM or PBS in both the early and late intervention groups (Figure 4a–c).

We conducted immunohistochemical staining for molecular markers to analyze the changes in plaque composition. In the early intervention group, although no statistically significant difference was observed, IL‐10M treated mice exhibited a higher expression of IL‐10 in their plaques (Figure 4a–c). In the late intervention group, IL‐10M treated mice demonstrated significantly elevated expression of IL‐10 in their plaques compared to PBS treated mice (Figure 4a–c). The results indicated no discernible differences in CD68(+) macrophages, smooth muscle cells, or collagen among the different treatment groups. However, there was a significant reduction in **MMP9**, a biomarker associated with plaque rupture, in the IL‐10M‐treated plaques from both the early and late intervention groups (Figure 4a–c).

Additionally, through dual fluorescence labeling of TUNEL and CD68 in the plaques of each group, we observed that the phagocytic index within IL‐10M‐treated plaques was similar to that in the ConM‐treated group. This suggests the preserved functionality of IL‐10M in clearing apoptotic cells within the plaque (Figure 4d–f).

### 2.5 Safety evaluation of engineered macrophage treatment

**FIGURE 5:**
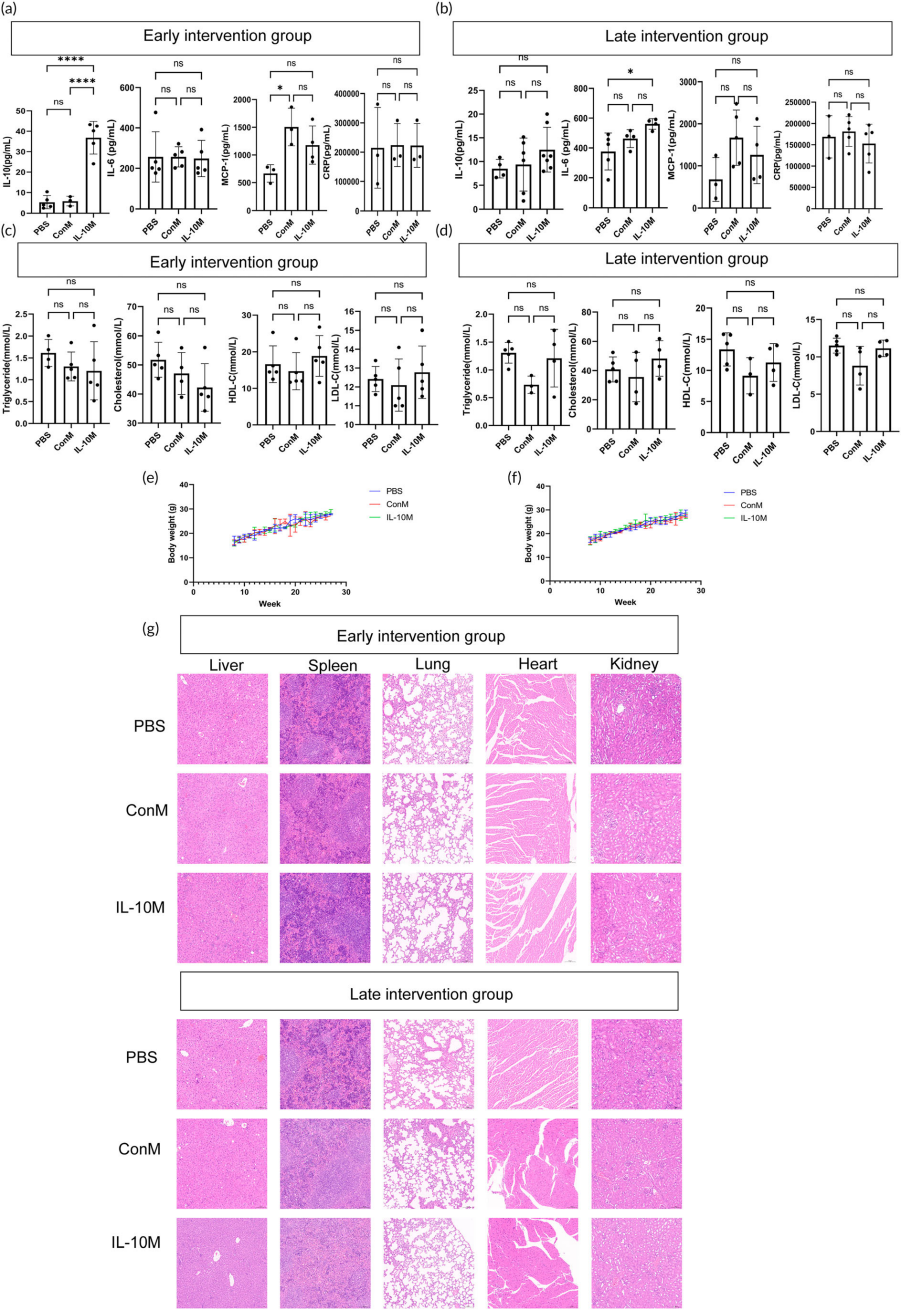
Safety validation of IL‐10M for treating atherosclerosis in mice. (a). After completing the treatment in the early intervention group, serum concentrations of IL‐10, IL‐6, MCP1, and CRP were measured. (b) After completing the treatment in the late intervention group, serum concentrations of IL‐10, IL‐6, MCP1, and CRP were measured. (c) After completing the treatment in the early intervention group, serum concentrations of triglycerides (TG), cholesterol (CHOL), HDL‐C, and LDL‐C were measured. (d) After completing the treatment in the late intervention group, serum concentrations of TG, CHOL, HDL‐C, and LDL‐C were measured. (e) Changes in body weight were observed among mice in the early intervention group. Data are presented as the mean ± SD. (f) Changes in body weight were observed among mice in the late intervention group. (g) H&E staining was performed on the heart, liver, spleen, lung, and kidney tissue sections after treatment in both the early and late intervention groups. Scale bar: 100 μm.

Despite the substantial therapeutic benefits of IL‐10M treatment, it did not result in evident side effects. We assessed cytokines (IL‐10, IL‐6, MCP1, and CRP) and blood lipids (triglyceride, cholesterol, HDL‐C, and LDL‐C) in the serum after treatment. Our findings revealed that IL‐10M‐treated mice exhibited an elevation of IL‐10 levels in the early intervention group and an increase of IL‐6 levels in the late intervention group (Figure 5a–d). Throughout the treatment period, we regularly monitored the weight of the mice. The weight gain patterns were consistent across the treatment groups (Figure 5e,f). We conducted post‐treatment organ assessments of the heart, liver, lungs, kidneys, and spleen, and histological analysis showed no significant differences (Figure 5g).

## 3 DISCUSSION

We first examined the effect of macrophage‐based therapy on reducing atherosclerotic burden. This therapy not only exhibits high targeting efficiency of macrophages but also enhances the effect of anti‐inflammation protein with short half‐lives. Our engineered macrophages overexpressing IL‐10 demonstrated an anti‐inflammatory M2 phenotype while maintaining high mitochondrial activity and normal phagocyte function. These cells exhibited the capability to (1) selectively target the aortic plaque area through tail vein injection, (2) significantly reduces plaque area and (3) decrease necrotic cores and MMP9 expression within plaques following both early and late interventions. Importantly, their administration did not induce any major organ dysfunction or weight abnormalities.

Inflammation plays a key role in the process of atherosclerosis development. Initially, low‐density lipoprotein (LDL) accumulates beneath the endothelium and undergoes various modifications, resulting in the formation of oxLDL, which triggers local inflammation.^21^ This leads to the recruitment of circulating monocytes to the damaged endothelium, where they differentiate into inflammatory macrophages and release various inflammatory factors.^22^ The recruitment of monocytes further fuels chronic inflammation and progression.^23^ Macrophages at the plaque site ingest oxLDL to form foam cells. The accumulation of lipids within these foam cells increases the likelihood of apoptosis, causing cell rupture and the subsequent release of lipids.^24^ This exacerbates inflammation and gradually weakens the plaque structure by reducing the collagen content and thinning the fibrous caps.^25^ Ultimately, plaque rupture can lead to acute thrombosis. Hence, the therapeutic potential of genetically engineered macrophages, designed based on their precise targeting ability, is noteworthy. This technology could provide a platform to capitalize on the intrinsic specificity of myeloid cell homing to the earliest sites of atherosclerosis when the lesions might be most susceptible to reversal.

Using viral infection methods, we genetically modified macrophages to overexpress the anti‐inflammatory cytokine IL‐10, thereby enabling its extracellular secretion without compromising their physiological functions. This modification can allow these cells to counteract inflammation at lesion sites upon arrival. Owing to their responsiveness to endogenous chemotactic factors, macrophages offer advantages for targeted therapy. Furthermore, their natural composition enhances their biocompatibility and simplifies their acquisition and construction compared with synthetic materials. Research has shown that macrophages with therapeutic effects can be obtained by inducing them to engulf specific drugs.^19^, ^26^ However, the inherent phagocytosis of drug particles by macrophages poses challenges, such as low loading efficiency, premature drug release, and undesirable drug inactivation. To address these issues, researchers have employed physicochemical methods, such as combining drug nanoparticles with macrophage membranes. For instance, Cheng and Li developed a biomimetic “core‐shell”‐structured nanoparticle comprising a PLGA core and a macrophage membrane shell.^20^ This design, leveraging the high affinity between α4β1 integrin and VCAM‐1 in the macrophage membrane, retains both the homing ability of macrophages and ensures high drug loading efficiency. However, the extraction of cell membranes and construction of suitable nanoparticles entail higher costs and technical requirements, impeding clinical translation. In our study, the engineered macrophages not only maintained complete homing ability but also preserved essential functions, such as phagocytosis and efferocytosis, which are crucial for the stability of atherosclerotic plaques. Importantly, the construction of engineered macrophages using lentiviruses was easily implemented.

In addition to their role as drug carriers, engineered macrophages have garnered attention for their ability to influence the microenvironment within the lesion area. Myeloid cells have been extensively studied and developed as targeted cell therapy tools against tumors. Within the tumor microenvironment, macrophages undergo reprogramming that diminishes their anti‐tumor abilities. However, modified macrophages can be endowed with anti‐tumor capabilities and regulate the tumor microenvironment. For instance, sustained expression of IFNβ in macrophages significantly inhibits the growth of intraperitoneal disseminated NUGC‐4 human gastric tumor in mice.^27^ Conversely, injections with non‐edited macrophages induces more pronounced tumor growth, suggesting that the tumor microenvironment may reprogram these cells back to a pro‐tumorigenic phenotype.^27^ Furthermore, the impact of genetically edited macrophages on the microenvironment is likely achieved through modulation of other immune cell functions. Genetically engineered myeloid cells expressing IL‐12 can reverse the immunosuppressive environment developed during metastatic progression by augmenting T cell responses and reducing metastatic burden in preclinical models.^28^ In the context of atherosclerosis, microenvironmental influence on macrophage function has also received considerable attention. Macrophages exhibit significant differences of phenotype and crosstalk based upon their microenvironment.^29^ Thus, it is crucial to implement modifications that have the potential to enhance the plaque stability.

Elevated serum IL‐10 levels have been linked to a more favorable prognosis in patients with acute coronary syndrome.^8^ Additionally, the TNF‐α:IL‐10 ratio was notably higher in patients with unstable angina compared to control patients. Exogenous IL‐10 significantly reduced TNF‐α and MMP9 levels in peripheral blood mononuclear cells of patients with unstable angina, suggesting that a decreased serum level of IL‐10 could be a marker of plaque instability and predict a poor prognosis after an acute ischemic event.^30^ Macrophages, which are crucial cellular sources of IL‐10, secrete IL‐10 to attenuate inflammation by suppressing the production of pro‐inflammatory cytokines.^31^, ^32^ Furthermore, IL‐10 can enhance the expression of anti‐inflammatory genes in cells, typically via a STAT3‐dependent pathway.^33^ In line with our results, IL‐10M exhibit an anti‐inflammatory phenotype and secrete more anti‐inflammatory factors. Within atherosclerotic plaques, excessive nitric oxide production causes a reaction with other reactive oxygen species, like superoxide anions, resulting in peroxynitrite formation, a strong oxidizing agent.^34^ Peroxynitrite can interact with biological molecules, such as lipids, DNA, and proteins, causing oxidative damage to cells and injuring endothelial tissues. Our findings suggest that IL‐10 overexpression endows macrophages with the ability to sustain reduced superoxide production and increased mitochondrial activity in inflammatory environments. After administration of IL‐10M into ApoE^−/−^ mice, a significant reduction in the levels of MMP9 was observed within aortic plaques. MMP‐9 can degrade the extracellular matrix including collagen. If the increased collagen degradation activity exceeds collagen synthesis, the fibrous cap gets thinner and becomes prone to rupture.^35^ IL‐10M stabilize the plaque microenvironment by maintaining their own activity, which may be the potential mechanism through which IL‐10M delay plaque progression.

Atherosclerosis frequently begins early in life, and its progression is accelerated by exposure to risk factors during childhood and adolescence.^36^ Mendieta et al. suggested that atherosclerosis regression is possible in the early stages of the disease. Utilizing multi‐area three‐dimensional vascular ultrasound imaging, a cohort of 3471 participants aged 40–55 years was longitudinally examined over a period of 6 years. Notably, 8% of patients with atherosclerotic plaque experienced complete plaque disappearance, with non‐smoking emerging as the strongest predictor for such a positive outcome.^36^ Therefore, early intervention in plaque progression can yield promising outcomes. In our study, IL‐10M treated mice in early intervention group exhibited a significant reduction in both the total area of aortic plaques and the ratio of plaque area to lumen area. However, while there was a notable decrease in the total plaque area of IL‐10M treated mice within the late intervention group, no significant reduction was observed in the ratio of plaque area to lumen area. A likely explanation of this result involves the concept of “trained immunity”, which suggests that repeated exposure to irritative stimuli may result in exaggerated responses.^37^ In mice fed a WD for up to 5 months, although the diet only caused transient systemic inflammatory responses, it led to long‐lasting changes in myeloid cell responses toward different innate immune stimuli.^38^ Our findings suggest that early anti‐inflammatory intervention may reduce plaque area by alleviating immune system reprogramming induced by the WD and mitigating MMP9 accumulation in plaques. However, when the intervention is conducted after 2 months of WD stimulation, the immune system may have already undergone long‐lived transcriptional and epigenetic reprogramming, resulting in a less pronounced efficacy of the anti‐inflammatory intervention initiated at this time. Histologically, plaques in the early stages of life are mainly classified as Type I–III (Type I Initial lesion, Type II Fatty streak, and Type III Intermediate lesion). These types of plaques exhibit a relatively uniform composition and less accumulation of inflammation, rendering them more likely to regress with intervention.^39^, ^40^ Therefore, continuous intervention with IL‐10M since the beginning of WD feeding can effectively promote regression of these early‐stage plaques. This regression can be observed both macroscopically and microscopically as a reduction in plaque area accompanied by a decrease in necrotic core size. However, after 2 months on WD feeding, most plaques may progress into advanced lesions (Type IV atheroma lesion, Type V fibroatheroma lesion, and Type VI complicated lesion), which possess a more complex composition and greater accumulation of inflammation.^39^, ^41^ These advanced stage plaques are less prone to regression but can still benefit from active intervention with IL‐10M by reducing necrotic cores and suppressing MMP9 expression to stabilize the plaque. Therefore, whether intervening with IL‐10M in early or late stages of life is beneficial for delaying plaque progression depends on different aspects. Early intervention effectively reduces overall plaque burden; whereas late‐stage intervention primarily focuses on stabilizing advanced lesions. Regardless of when intervention commences, IL‐10M holds potential as an effective medication for reducing cardiovascular events and alleviating the national health burden caused by CVD.

Currently, additional investigation is necessary to translate and evaluate the efficacy of this approach in humans. In this study, IL‐10M was built with RAW264.7, an immortalized cell line derived from mice, which exhibited excellent biocompatibility with ApoE^−/−^ C57BL/6 mice and demonstrated robust proliferation ability and controllable quality. These characteristics make the cells suitable for the development of treatment strategies. Our vision for this platform in patients with atherosclerosis is to collect cells up front for IL‐10M production and then reintroduce them back into the patient. The safety and efficacy of similar approaches have been demonstrated in clinical trials conducted for diverse diseases.^42^, ^43^, ^44^ In addition, IL‐10M can also be designed to deliver agents that demonstrate limited benefit when administered systemically and factors that precisely target other mechanism mediating inflammatory niche formation and progression within atherosclerotic plaques. Furthermore, in addition to ApoE^−/−^ mice, Ldlr^−/−^ mice are commonly used to model atherosclerosis, and the properties of lipoproteins and mechanisms promoting atherosclerosis differ from those of ApoE^−/−^ mice. To address the heterogeneity of human pathogenesis, it is imperative to validate with diverse animal models constructed based on various principles. Moreover, an inducible expression system and/or turnoff strategy should be developed and implemented in future investigations to minimize side effects during modified cell migration.

## 4 MATERIALS AND METHODS

### 4.1 Cell culture

RAW264.7 and HEK293T cells were cultured in RPMI 1640 (Gibco) supplemented with 10% (v/v) fetal bovine serum (FBS; Gibco), 100 μg/ml penicillin, and 100 μg/ml streptomycin.

### 4.2 Establishing engineered macrophages

Lentiviral constructs expressing murine IL‐10 were cloned into a pCDH‐GFP‐pure vector. The lentiviral vectors were transfected with packing plasmids into 293 T cells, and the viral particles were collected and used to infect RAW264.7 cells. Cells infected with IL‐10 loaded vectors were labeled as IL‐10M, while the cells infected with empty vectors were labeled as ConM. Selection was conducted by culturing the cells in a medium containing 5 μg/ml puromycin for 2 days.

### 4.3 Mouse model

Six‐week‐old female ApoE^−/−^ mice were fed a Western diet (WD) containing 21% fat, 50% carbohydrate, 20% protein, and 0.21% cholesterol to induce atherosclerosis. At the end of the treatment, the mice were euthanized, and the degree of pathological changes was evaluated by measuring the lesion area of the aorta from the heart to the iliac bifurcation after Oil Red O (ORO) staining. All animal experiments were approved by the Experimental Animal Ethics Committee at the Chinese PLA General Hospital (approval number: 2022‐X18‐109).

### 4.4 ELISA

The levels of cytokines in the supernatant of the medium and the serum from the mice were measured using commercially available mouse IL‐10, MCP‐1, IL‐6, CRP, and TNF‐α ELISA kits (Servicebio, Wuhan, China). The experiment was repeated thrice, and cytokine concentrations were calculated using standard curves.

### 4.5 RT‐qPCR analysis

mRNA was extracted using the RNAsimple Total RNA Kit (TIANGEN, Beijing, China), following the manufacturer's protocol. Reverse transcription was performed using the ReverTra Ace qPCR RT Master Mix (Toyobo, Osaka, Japan). cDNA was mixed with the SYBR Green Real‐Time PCR RT Master Mix (Toyobo) to perform RT‐qPCR. All PCRs were run at least in triplicate, and target RNA expression was normalized to β‐actin levels. Relative expression was calculated by normalizing to the control samples using the 2^−∆∆Ct^ method. The sequences of the PCR primers used are as follows:

Cd206: forward 5′‐CTCTGTTCAGCTATTGGACGC‐3′.

Reverse 5′‐TGGCACTCCCAAACATAATTTGA‐3′.

Arg1: forward 5′‐CTGGGGATTGGCAAGGTGAT‐3′.

Reverse 5′‐CAGCCCGTCGACATCAAAG‐3′.

Tnf‐α: forward 5′‐AGCCGATGGGTTGTACCTTG‐3′.

Reverse 5′‐ATAGCAAATCGGCTGACGGT‐3′.

Inos: forward 5′‐AGCTCGGGTTGAAGTGGTATG‐3′.

Reverse 5′‐CACAGCCACATTGATCTCCG‐3′.

β‐actin: forward 5′‐GGCTGTATTCCCCTCCATCG‐3′.

Reverse 5′‐CCAGTTGGTAACAATGCCATGT‐3′.

### 4.6 Bulk RNA sequencing and analysis

IL‐10M and control cells ConM were treated with 100 ng/ml lipopolysaccharide (LPS; Sigma‐Aldrich, L2654, Shanghai, China) for 12 h, 20 μg/ml oxidized low‐density lipoprotein (oxLDL) (Solarbio, Beijing, China) for 24 h, or the vehicle (PBS). RNA was extracted using TRIzol (Magen). PE libraries were prepared according to the instructions for the ABclonal mRNA‐seq Lib Prep Kit (ABclonal, Wuhan, China). The library quality was evaluated using an Agilent Bioanalyzer 4150. Sequencing was performed using a NovaSeq 6000 platform with a PE150 read length. The data generated from the Illumina platform were used for subsequent analyses. We used DESeq2 v3.17 for differential gene expression analysis. A default *p* value <0.05 was considered statistically significant, with a fold‐change ≥2 for up‐regulated transcripts or ≤−2 for down‐regulated transcripts. We created a list of differentially expressed genes that were used to generate volcano plots and perform Kyoto Encyclopedia of Genes and Genomes (KEGG) pathway analysis and gene set enrichment analysis (GSEA) using the clusterProfiler R v4.2 package. Raw and normalized data are accessible under the NCBI Gene Expression Omnibus and through the GEO Series accession number GSE248791.

### 4.7 Flow cytometry analysis

Engineered RAW264.7 cells were washed with PBS and resuspended in 0.5% bovine serum albumin in PBS. The cells were incubated in antibody dilutions to detect CD206 (BioLegend, San Diego, CA), CD86 (BioLegend), F4/80 (BioLegend), and CD11b (BioLegend) according to the manufacturer's instructions. Data were acquired using FACSCanto II (BD, Franklin Lakes, NJ), and further analyses were performed using FACSDiva (version 8.0.2; BD).

### 4.8 MitoSOX and MitoTracker analysis

IL‐10M and ConM were plated in RPMI 1640 supplemented with 10% FBS and treated with 100 ng/ml LPS for 12 h and oxLDL for 24 h. After treatment, the cells were washed twice with cold PBS, harvested, and stained with MitoSOX Red Mitochondrial Superoxide Indicator (Wobo, Nanjing, China) and MitoTracker Red CMXRos (Solarbio, Beijing, China) according to the manufacturer's instructions. For flow cytometry analysis, data were acquired using FACSCanto II (BD, Franklin Lakes, NJ) and analyzed using FACSDiva (version 8.0.2; BD).

### 4.9 In vitro efferocytosis assay

Standard in vitro efferocytosis assays were performed using RAW 264.7 macrophages as target cells. Apoptosis in target cells was induced using 1 μM staurosporine (Macklin, Shanghai, China) for 4 h at 37°C. Target cells were labeled using the PKH26 Red Cell Membrane Staining Kit (Solarbio, Beijing, China) according to the manufacturer's instructions. IL‐10M and ConM spontaneously emit green fluorescence. IL‐10M or ConM were co‐cultured with targeted cells for 2 h at 37°C. The efferocytosis rate was calculated by dividing the number of double‐positive cells by the total number of apoptotic cells. For quantitative flow analysis, data were acquired using FACSCanto II (BD, Franklin Lakes, NJ) and analyzed using FACSDiva (version 8.0.2; BD).

### 4.10 ORO staining in macrophages

IL‐10M and ConM were treated with oxLDL (20 μg/ml; Solarbio, Beijing, China) for 24 h, and the cells were washed thrice with PBS. Subsequently, the macrophages were stained with freshly prepared ORO working solution for 15 min and rinsed with 60% isopropanol. After washing thrice with PBS, the nuclei of the macrophages were slightly stained with DAPI (Beyotime, Beijing, China). Finally, the macrophages were washed with PBS and examined under a microscope. The lipid phagocytosis index was defined as the ratio of the lipid droplet area to the number of cells in the region of interest.

### 4.11 Serum biochemistry

Blood samples were collected after fasting for 8 h. The whole blood samples were kept at 25°C for 2 h and centrifuged at 4°C for 3000×*g* for 15 min. Then, the supernatants were stored at −80°C for detection. Blood lipid levels were measured using a Chemray 800 chemistry analyzer and its reagents (Rayto, Shenzhen, China).

### 4.12 In vivo targeting of the atherosclerotic plaques

Eight‐week‐old female ApoE^−/−^ mice fed with WD (with the same composition as described above) for 3 month were intravenously administered IL‐10M via the tail vein. After allowing distribution for 6 h, the mice were euthanized. The aortas were isolated for imaging using the intravital imaging system (IVIS; Lumina XR III, PerkinElmer, Waltham, MA) and analyzed using the Living Image software (Version 4.4; PerkinElmer).

### 4.13 In vivo therapeutic efficacy study

First, the ApoE^−/−^ mice were divided into two groups based on the timing of the intervention. The early intervention group (*n* = 45) received intravenous tail injections from the beginning of WD at 6 weeks old. The late intervention group (*n* = 45) started receiving injections at 16 weeks old, 2 months after being fed the WD. Depending on the injection content, the mice were further divided into IL‐10M, ConM, and PBS groups. The early intervention group received injections every 2 weeks and the late intervention group received injections once a week. Both groups stopped receiving injections after 5 months of being fed the WD. Mouse aortas were stained with ORO to label plaques and were photographed for quantitative analysis using the ImageJ (v6.0; NIH) software. Throughout the process, the weight and survival status of the mice were continuously observed and recorded. The hearts, livers, spleens, lungs, and kidneys of the mice were sectioned for hematoxylin and eosin (H&E) staining to observe morphological changes.

### 4.14 Histological study regarding aortic tissues

For histological analysis, the aorta from mice subjected to various treatments were collected for H&E and Masson's trichrome staining and immunohistochemistry analysis with antibodies, namely CD68, **MMP9**, α‐SMA, and IL‐10 (Proteintech, Wuhan, China).

### 4.15 Analysis of lesional efferocytosis

In situ efferocytosis was performed using mouse aortic tissues as previously described. The aortic sections were dual‐stained with TUNEL and CD68 antibodies (Servicebio, Wuhan, China). The nuclei were stained with DAPI (Beyotime, Beijing, China). We considered free apoptotic cells those TUNEL‐positive nuclei that do not overlap with CD68, whereas TUNEL‐positive nuclei that do overlap with CD68 were considered phagocytosed apoptotic cells. Pictures of three consecutive sections of the aorta were used for quantification. The results are presented as the ratio of phagocytosed to free apoptotic cells.

### 4.16 Statistical analysis

Comparisons between two groups were performed using Student's *t*‐test. Comparisons between multi‐groups were performed using one‐way ANOVA or the Kruskal–Wallis test based on the data's distribution and followed by post hoc analysis using Tukey's multiple comparisons test or Dunnet's test. Values of *p ≤ 0.05, **p ≤ 0.01, ***p ≤ 0.001, and ****p ≤ 0.0001 were used to indicate statistical significance. All data are presented as mean values ± the standard deviations or median ± the interquartile range of independent experiments.

## 5 CONCLUSIONS

We developed an anti‐inflammatory protein delivery system based on engineered macrophages that can target atherosclerotic plaques and reduce the plaque area and necrotic core. This approach has a promising safety profile and has the potential to be a therapeutic strategy for the intervention of atherosclerosis.

## AUTHOR CONTRIBUTIONS

**Mingyi Wang:** Conceptualization; investigation; methodology; writing – original draft; writing – review and editing. **Shanshan Zhou:** Conceptualization; investigation; methodology; writing – original draft; writing – review and editing. **Yingyun Hu:** Investigation; methodology. **Wei Tong:** Conceptualization; methodology. **Hao Zhou:** Investigation; methodology. **Mingrui Ma:** Investigation; methodology. **Xingxuan Cai:** Investigation; methodology. **Zhengbin Zhang:** Investigation; methodology. **Luo Zhang:** Conceptualization; project administration; resources; supervision; writing – review and editing. **Yundai Chen:** Conceptualization; methodology; project administration; resources; supervision; writing – review and editing.

## FUNDING INFORMATION

No funding was received for this study.

## CONFLICT OF INTEREST STATEMENT

The authors declare no conflicts of interest.

## Supporting information


**FIGURE S1.** Fluorescence imaging of major organs after single injection in mice. Fluorescence imaging of major organs was performed in ApoE^−/−^ mice modeled with WD for 3 months, and injected with PBS, ConM, or IL‐10M at a dose of 1 × 10^7^ cells. The heart, liver, spleen, lungs, and kidneys were imaged ex vivo at 6 and 72 h post‐injection.
**FIGURE S2.** Fluorescence imaging of liver and spleen pathological slices following a single injection. Fluorescence imaging of major organs was performed in ApoE^−/−^ mice modeled with WD for 3 months, and injected with PBS, ConM, or IL‐10M at a dose of 1 × 10^7^ cells. Liver and spleen tissues were collected from the mice at both 6 and 72 h post‐injection for fluorescence imaging analysis. Scale bar: 50 μm.
**FIGURE S3.** Concentration of IL‐10 in major organs and serum following a single injection. (a) ApoE mice feed with WD for a duration of 3 months were intravenously injected with PBS, ConM, and IL‐10M. The cell injection dosage was 1 × 10^7^. After 6 and 72 h post‐injection, the concentrations of IL‐10 in mouse heart, liver, spleen, lung, and kidney tissues were quantified. (b) ApoE mice feed with WD for a duration of 3 months were intravenously injected with PBS, ConM, and IL‐10M. The cell injection dosage was 1 × 10^7^. After 6 and 72 h post‐injection, the levels of IL‐10 in mouse serum were measured. Comparisons between multi‐groups were performed using one‐way ANOVA. Values of *p ≤ 0.05, **p ≤ 0.01, ***p ≤ 0.001, were used to indicate statistical significance.
**FIGURE S4.** H&E staining of major organs following a single injection. ApoE mice feed with WD for a duration of 3 months were intravenously injected with PBS, ConM, and IL‐10M. The cell injection dosage was 1 × 10^7^. After 6 and 72 h post‐injection, liver, spleen, lung, and kidney were collected for histopathological sectioning and H&E staining. Scale bar: 100 μm.
**FIGURE S5.** Aorta from mice of early and late intervention group before first cell injection. Oil Red O staining was performed on the aortas of 8‐week‐old ApoE^−/−^ mice without WD feeding and 16‐week‐old ApoE^−/−^ mice with an 8‐week WD feeding.

## Data Availability

All data presented in the main text are available from the corresponding authors upon request.
