# Supplementary material for: Macrophages overexpressing interleukin‐10 target and prevent atherosclerosis: Regression of plaque formation and reduction in necrotic core
Source: Bioeng Transl Med. 2024 Sep 16;10(1):e10717. doi: 10.1002/btm2.10717 (PMC11711221; doi:10.1002/btm2.10717)
Supplement: Supplementary file 1 — FIGURE S1. Fluorescence imaging of major organs after single injection in mice. Fluorescence imaging of major organs was performed in ApoE−/− mice modeled with WD for 3 months, and injected with PBS, ConM, or IL‐10M at a dose of 1 × 10^7^ cells. The heart, liver, spleen, lungs, and kidneys were imaged ex vivo at 6 and 72 h post‐injection. FIGURE S2. Fluorescence imaging of liver and spleen pathological slices following a single injection. Fluorescence imaging of major organs was performed in ApoE−/− mice modeled with WD for 3 months, and injected with PBS, ConM, or IL‐10M at a dose of 1 × 10^7^ cells. Liver and spleen tissues were collected from the mice at both 6 and 72 h post‐injection for fluorescence imaging analysis. Scale bar: 50 μm. FIGURE S3. Concentration of IL‐10 in major organs and serum following a single injection. (a) ApoE mice feed with WD for a duration of 3 months were intravenously injected with PBS, ConM, and IL‐10M. The cell injection dosage was 1 × 10^7^. After 6 and 72 h post‐injection, the concentrations of IL‐10 in mouse heart, liver, spleen, lung, and kidney tissues were quantified. (b) ApoE mice feed with WD for a duration of 3 months were intravenously injected with PBS, ConM, and IL‐10M. The cell injection dosage was 1 × 10^7^. After 6 and 72 h post‐injection, the levels of IL‐10 in mouse serum were measured. Comparisons between multi‐groups were performed using one‐way ANOVA. Values of *p ≤ 0.05, **p ≤ 0.01, ***p ≤ 0.001, were used to indicate statistical significance. FIGURE S4. H&E staining of major organs following a single injection. ApoE mice feed with WD for a duration of 3 months were intravenously injected with PBS, ConM, and IL‐10M. The cell injection dosage was 1 × 10^7^. After 6 and 72 h post‐injection, liver, spleen, lung, and kidney were collected for histopathological sectioning and H&E staining. Scale bar: 100 μm. FIGURE S5. Aorta from mice of early and late intervention group before first cell injection. Oil Red O staining was perf [file BTM2-10-e10717-s001.docx]

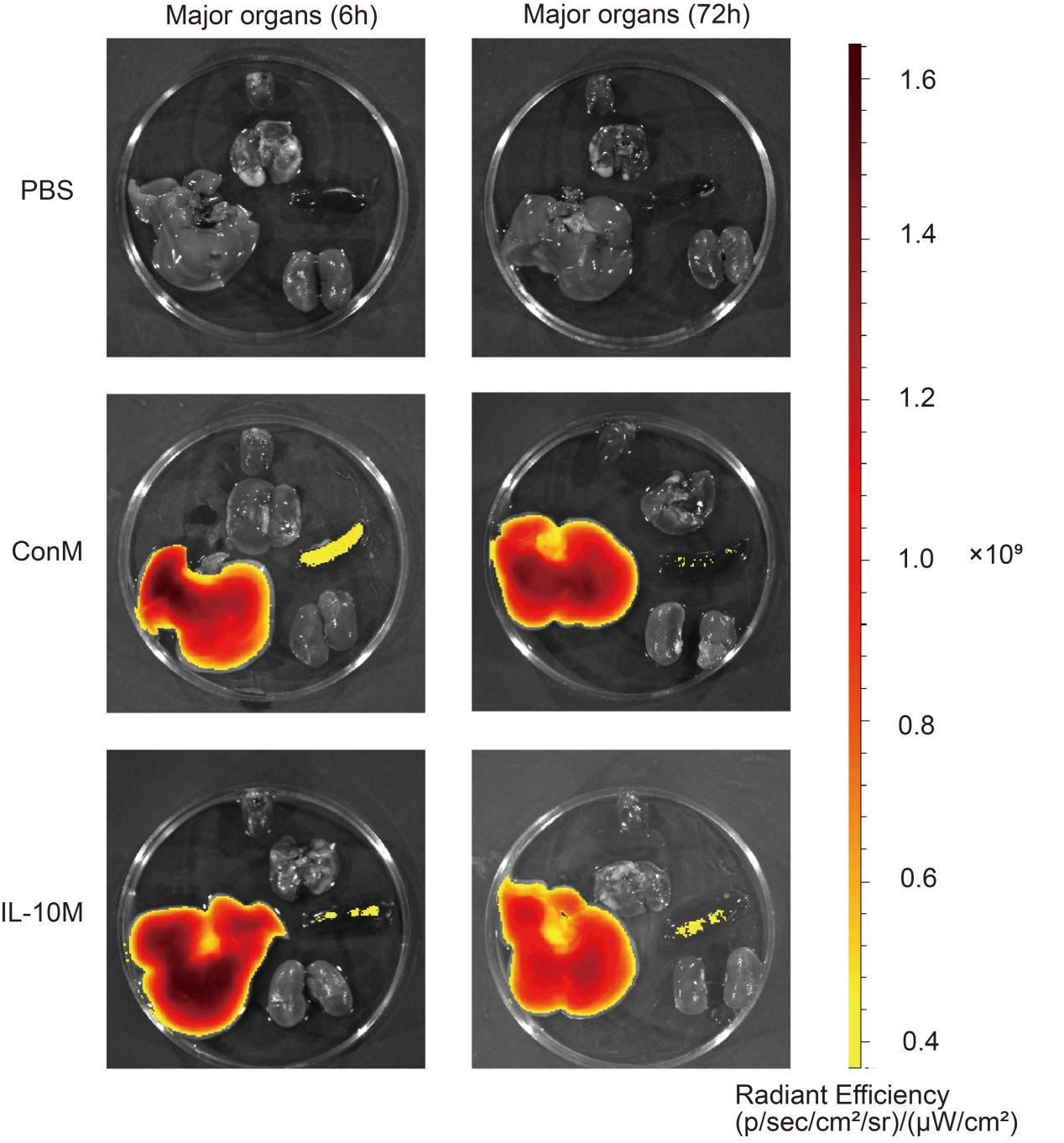


**Figure S1** Fluorescence imaging of major organs after single injection in mice

Fluorescence imaging of major organs was performed in ApoE-/- mice modeled with WD for 3 months, and injected with PBS, ConM, or IL-10M at a dose of 1×10^7^ cells. The heart, liver, spleen, lungs, and kidneys were imaged *ex vivo* at 6 and 72 h post-injection.


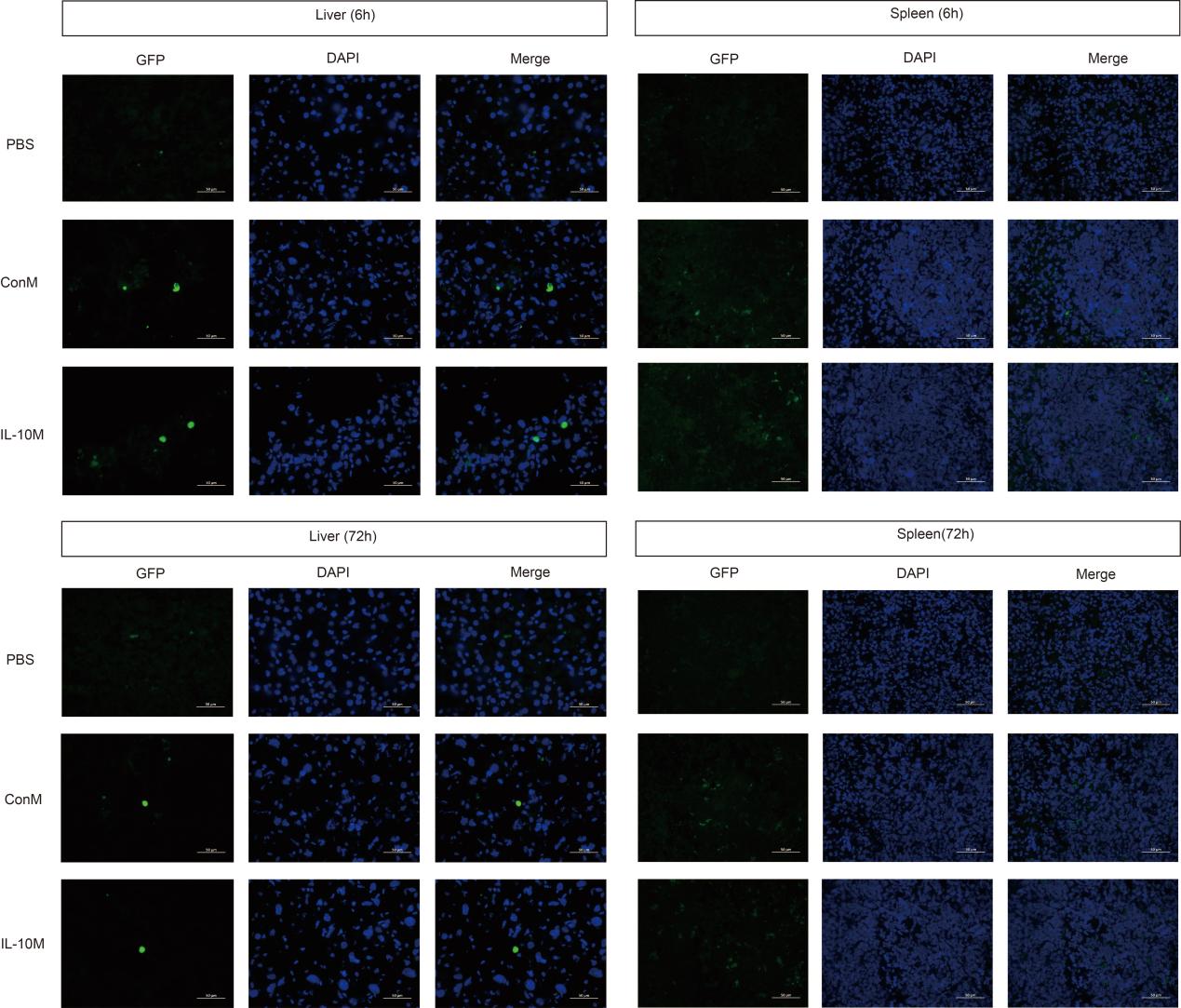


**Figure S2** Fluorescence imaging of liver and spleen pathological slices following a single injection. Fluorescence imaging of major organs was performed in ApoE-/- mice modeled with WD for 3 months, and injected with PBS, ConM, or IL-10M at a dose of 1×10^7^ cells. Liver and spleen tissues were collected from the mice at both 6 and 72 h post-injection for fluorescence imaging analysis. Scale bar: 50μm


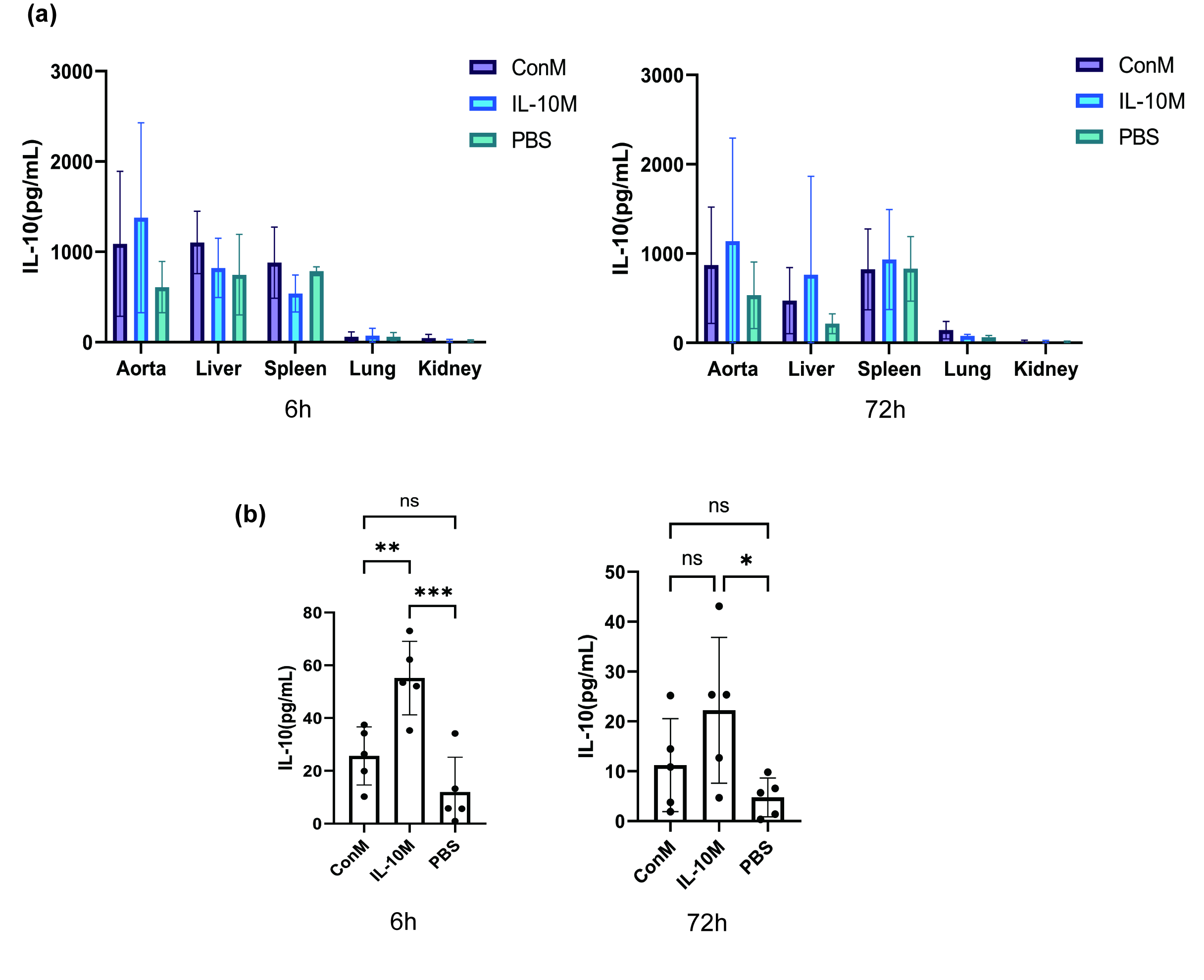


**Figure S3** Concentration of IL-10 in major organs and serum following a single injection

(a) ApoE mice feed with WD for a duration of 3 months were intravenously injected with PBS, ConM, and IL-10M. The cell injection dosage was 1×10^7^. After 6 and 72 h post-injection, the concentrations of IL-10 in mouse heart, liver, spleen, lung, and kidney tissues were quantified. (b) ApoE mice feed with WD for a duration of 3 months were intravenously injected with PBS, ConM, and IL-10M. The cell injection dosage was 1×10^7^. After 6 and 72 h post-injection, the levels of IL-10 in mouse serum were measured. Comparisons between multi-groups were performed using one-way ANOVA. Values of *P ≤ 0.05, **P ≤ 0.01, ***P ≤ 0.001, were used to indicate statistical significance.


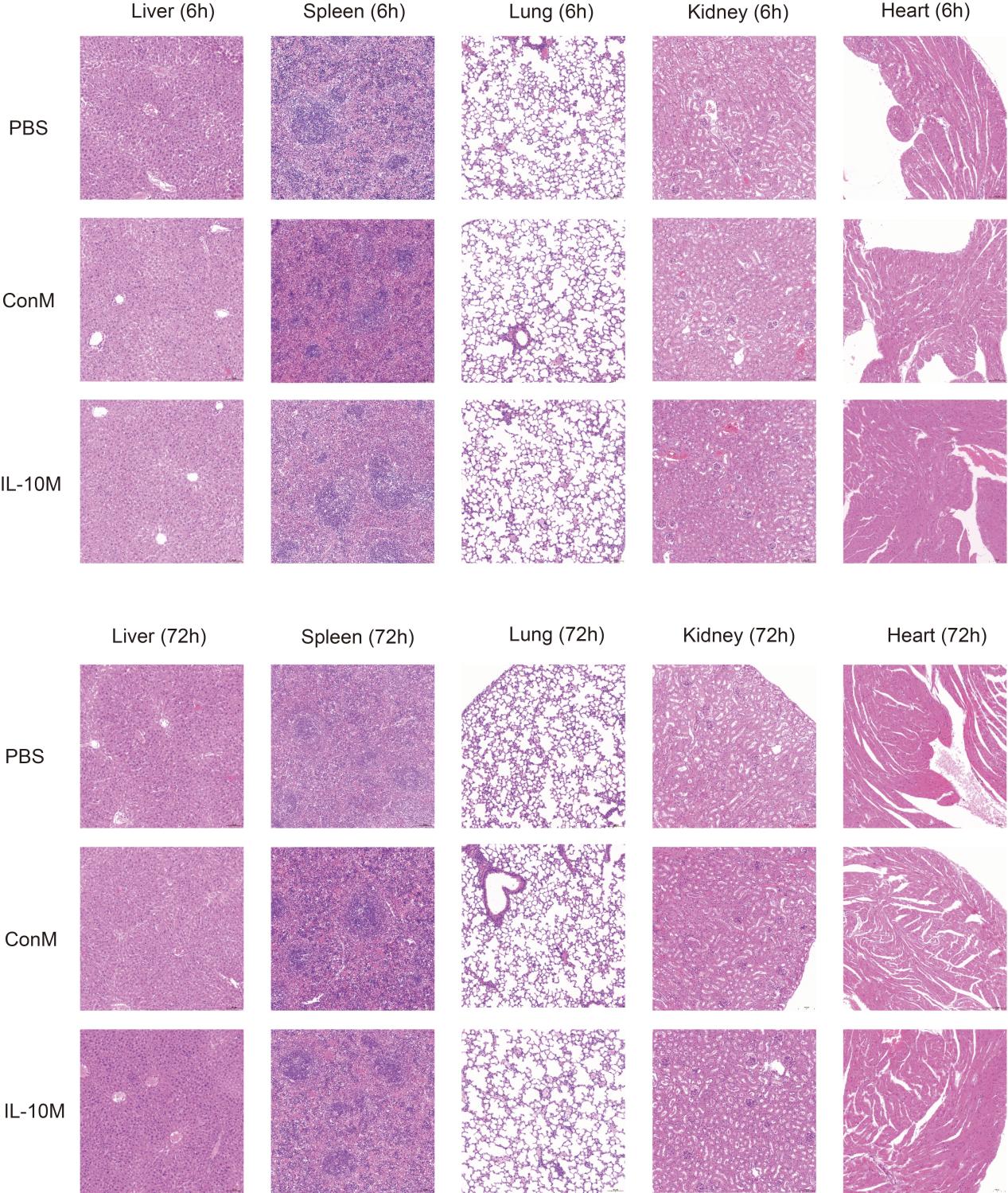


**Figure S4** H&E staining of major organs following a single injection

ApoE mice feed with WD for a duration of 3 months were intravenously injected with PBS, ConM, and IL-10M. The cell injection dosage was 1×10^7^. After 6 and 72 h post-injection, liver, spleen, lung, and kidney were collected for histopathological sectioning and H&E staining. Scale bar: 100 μm


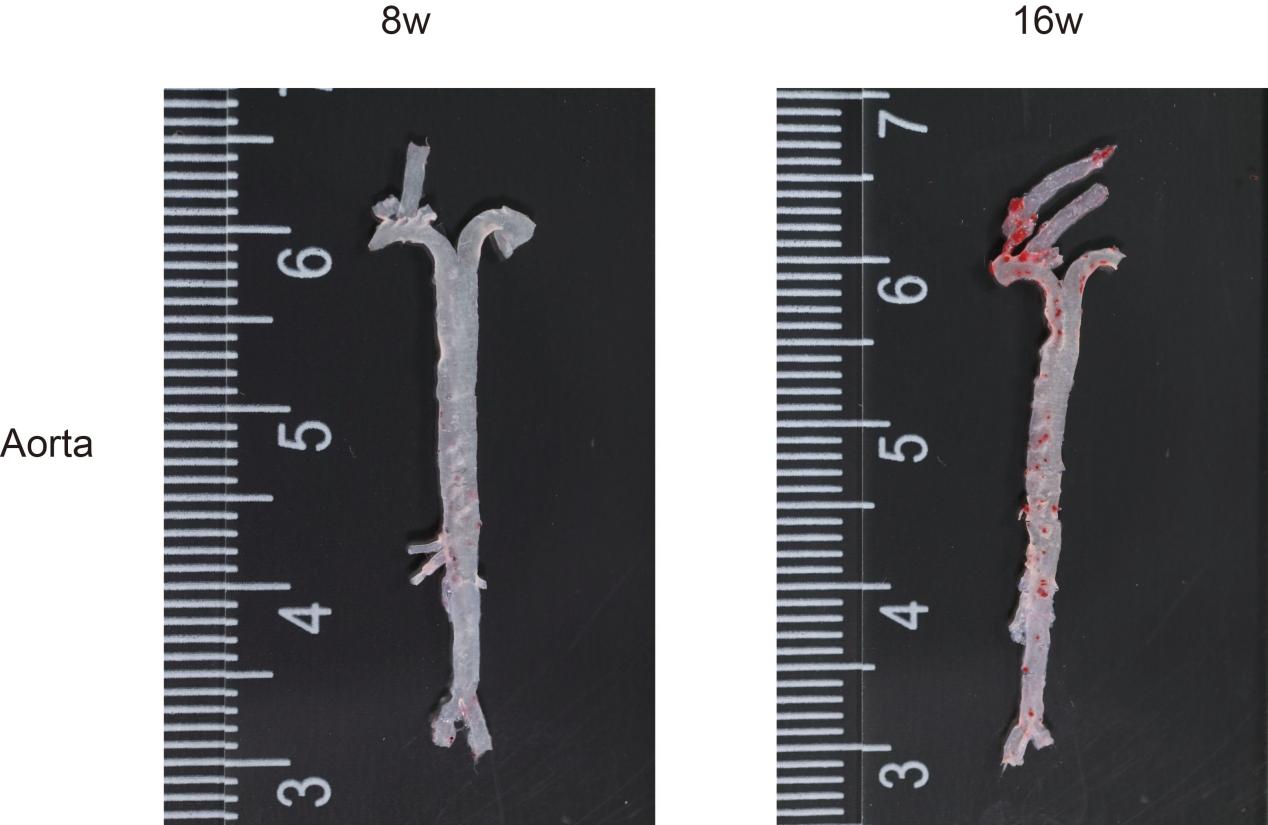


**Figure S5** Aorta from mice of early and late intervention group before first cell injection.

Oil Red O staining was performed on the aortas of 8-week-old ApoE-/- mice without WD feeding and 16-week-old ApoE-/- mice with an 8-week WD feeding.
